# Localization of nanoscale objects with light singularities

**DOI:** 10.1515/nanoph-2024-0639

**Published:** 2025-03-19

**Authors:** Thomas A. Grant, Anton N. Vetlugin, Eric Plum, Kevin F. MacDonald, Nikolay I. Zheludev

**Affiliations:** Optoelectronics Research Centre, University of Southampton, Southampton, SO17 1BJ, UK; Centre for Disruptive Photonic Technologies, SPMS & TPI, Nanyang Technological University, Singapore, 637371, Singapore; Hagler Institute for Advanced Study, Texas A&M University, College Station, Texas, 77843, USA

**Keywords:** superoscillation, singularities, nanophotonics; optical metrology

## Abstract

Unprecedented atomic-scale measurement resolution has recently been demonstrated in single-shot optical localization measurements based on deep-learning analyses of diffraction patterns of topologically structured light scattered from objects. Here, we show that variations in the diffraction patterns caused by positional changes of an object depend upon the spatial derivatives of the amplitude and phase of the incident field, most strongly around phase singularities. Despite lower intensity near the singularity, an orders-of-magnitude increase in Fisher information contained in the diffraction patterns can be achieved when a nano-object is illuminated by light containing phase singularities, rather than a plane wave. Our work provides a fundamental explanation and motivation for singularity-based metrology with deeply subwavelength precision.

## 1 Introduction

In recent decades, progress in optical super-resolution microscopy and metrology has been driven by nonlinear and statistical techniques [1], [2], [3], [4], [5], [6], [7], [8], [9], [10], [11], structured illumination microscopy [12], [13], [14], and computational imaging techniques for retrieving phase from scattered light [15], [16], [17], [18], [19], [20], [21], often taking advantage of object sparsity [22], [23], [24]. The ability of neural networks to efficiently solve the inverse scattering problem has also been demonstrated [25], and superoscillatory (topologically structured) light fields have lately been applied to microscopy and optical measurement applications in a manner similar to computational imaging.

The phenomenon of optical superoscillation was introduced [26] in 2006 and experimentally identified shortly thereafter [27]. It describes rapid subwavelength spatial variations of intensity and phase in complex electromagnetic fields formed by the interference of several coherent waves, and its observation stimulated a significant revision of the limits of classical electromagnetism. In particular, computational and experimental studies of the topological structure of superoscillatory fields in free space have revealed subdiffraction energy “hotspots” and high local wavevectors, facilitated by the presence of phase singularities bordering regions of energy backflow (i.e., powerflow vortices) [28], [29]. These can be orders of magnitude smaller than the wavelength, implying that their interaction with matter should vary on similarly short, subwavelength scales, which makes their application a promising prospect for metrology.

Berry and Nye proposed a form of singularity-based metrology in the 1970s, suggesting that singularities (referred to then as “wave dislocations”) in radio pulses reflected by the rock bed of a glacier could be employed as subwavelength markers for echo-sounding-based depth measurements [30], [31]. More recently, dimensional and positional measurements with deeply subwavelength resolution have been achieved via deep learning analysis of objects’ diffraction patterns [32], [33], [34]. With topologically structured illumination and “*in situ*” neural network training, such measurements can localize the average position of a nanowire with precision and accuracy down to ∼100 pm using visible light [35], [36], beating the diffraction limit of conventional optical instruments thousands of times over.

In this work, we mathematically describe and numerically demonstrate that the scattering from an object located near a singularity in a topologically structured field has higher information content than the scattered field from a plane wave, thereby enabling greater precision in measurements based upon its analysis (in estimation theory, Fisher information determines the upper limit of precision). We show that this advantage derives from the presence of strong phase and intensity variations over short length scales in the incident field (i.e., in the vicinity of singularities): for an archetypal single-slit diffraction configuration, Fisher information associated with a small positional shift of the slit in a superoscillatory incident field is enhanced by ∼250× (compared to a plane wave incident field), when a singularity is located near to the centre of the slit.

## 2 Theoretical analysis

We begin with the Rayleigh–Sommerfeld model of diffraction – a mathematical manifestation of the Huygens–Fresnel principle [37]. For simplicity in the present case, we reduce this to a two-dimensional form, whereby the scattered field is expressed as a superposition of diverging circular waves radiating from a 1D array of points describing the scattering object (along *x* at *z* = 0),

$$U\left(x\right)\propto i\int \tilde{U}\left(x^{\prime }\right)\frac{\exp\left(i2\pi r/\lambda \right)}{r}\cos\theta dx^{\prime }$$

where $\tilde{U}\left(x^{\prime }\right)$ and $U\left(x\right)$ denote the complex field of a monochromatic wave, with wavelength *λ*, at the object and the detector, respectively, which are separated by a distance *h* in the propagation direction *z*, whereby $r=\sqrt{{\left(x-x^{\prime }\right)}^{2}+h^{2}}$ , and $\theta =\arctan\left(\frac{x-x^{\prime }}{h}\right)$ .

As an archetypal scattering object, we consider a narrow slit in an otherwise opaque screen, with edges located at *x*′ = *a* ± *δ* (i.e., a slit of width 2*δ* centered at *x*′ = *a*). We assume that a complex field, $\tilde{U}\left(x^{\prime }\right)=A\left(x^{\prime }\right){\text{e}}^{\text{i}\phi \left(x^{\prime }\right)}$ , is normally incident on the screen and is transmitted only through the slit. Following integration by parts, we can write the scattered field, $U\left(x\right)$ at the detector as a sum of three contributions:

$$U\left(x\right)=U_{1}\left(x\right)+U_{2}\left(x\right)+U_{3}\left(x\right)$$

where

(1) $$\begin{array}{ll} U_{1}\left(x\right) & =A\left(a+\delta \right){\text{e}}^{\text{i}\phi \left(a+\delta \right)}\xi \left(x,a+\delta \right)\\ & \;-A\left(a-\delta \right){\text{e}}^{\text{i}\phi \left(a-\delta \right)}\xi \left(x,a-\delta \right) \end{array}$$

$$U_{2}\left(x\right)=-\overset{a+\delta }{\underset{a-\delta }{\int }}\frac{dA\left(x^{\prime }\right)}{dx^{\prime }}{\text{e}}^{\text{i}\phi \left(x^{\prime }\right)}\xi \left(x,x^{\prime }\right)dx^{\prime }$$

$$U_{3}\left(x\right)=-i\overset{a+\delta }{\underset{a-\delta }{\int }}\frac{\text{d}\phi \left(x^{\prime }\right)}{dx^{\prime }}A\left(x^{\prime }\right){\text{e}}^{\text{i}\phi \left(x^{\prime }\right)}\xi \left(x,x^{\prime }\right)dx^{\prime }$$

and

$$\xi \left(x,x^{\prime }\right)\propto i\int \frac{\exp\left(i2\pi r/\lambda \right)}{r}\cos\theta dx^{\prime }.$$

Here, *U*_1_ is the only term present in the diffracted field from an incident plane wave, while *U*_2_ and *U*_3_ are, respectively, dependent on variations in the amplitude and phase of the incident field over the scattering object. The changes in these additional contributions to the scattered field for a structured incident field, arising from changes in the object plane, can become significant in comparison to the associated change in *U*_1_. Thus, the spatially fast-changing features of a structured incident field can cause changes in *U*_2_ and *U*_3_ to dominate the total change in the scattered field.

## 3 Numerical methods

**Figure 1:**
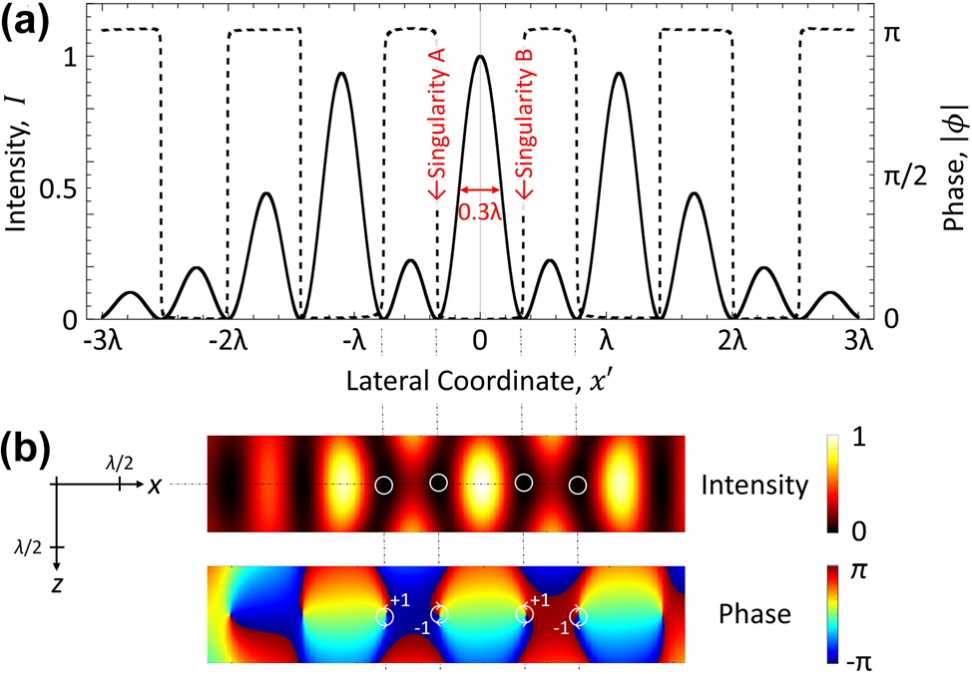
Superoscillatory field profile. Intensity $I\left(x^{'}\right)=\tilde{U}\left(x^{'}\right)\tilde{U}{\left(x^{\prime }\right)}^{*}$ [solid line], and corresponding phase $\phi \left(x^{\prime }\right)$ [dashed line] profiles, of the superoscillatory field $\tilde{U}\left(x^{\prime }\right)=\left[21.65S_{2}\left(x^{\prime }\right)+S_{3}\left(x^{\prime }\right)\right]W$ in the object plane (*z* = 0). (b) Maps of intensity and phase in the *xz* plane – phase singularities, at low intensity points in the former, are labeled with their topological charge values in the latter.

As a practically relevant example, following the methods described in Refs [38], [39], [40] and recent experimental work [32], [35], [36], we consider a superoscillatory field formed by the linear combination of two band-limited, prolate spheroidal wave functions (PSWFs): $\tilde{U}\left(x^{\prime }\right)=\left[21.65S_{2}\left(x^{\prime }\right)+S_{3}\left(x^{\prime }\right)\right]W$ , with *W* = 0.00021. While the two individual PSWFs are band-limited to $\left|k_{0}\right|=\omega /\text{c}$ , $\tilde{U}\left(x^{\prime }\right)$ has a central peak focused beyond this limit (to a full-width at half-maximum of 0.3*λ*), flanked by a series of singularities (Figure 1).

As detailed in Ref. [41], the phase and amplitude mask required to generate this superoscillatory field from a plane wave can be obtained by transforming the required object-plane field $\tilde{U}\left(x^{\prime }\right)$ into a Fourier series (PSWFs being eigenfunctions of a finite, band-limited Fourier transform); backpropagating to the desired mask plane; and then executing an inverse Fourier transform. Here, we assume a mask plane at a distance *d* = 30*λ* from the object plane, under which condition the intensity at the peak of the superoscillatory field’s central hotspot $U\left(x^{\prime }\right)U{\left(x^{\prime }\right)}^{*}$ is approximately twice (2.06×) the intensity of the plane wave incident upon the mask, $U_{0}U_{0}^{*}$ .

**Figure 2:**
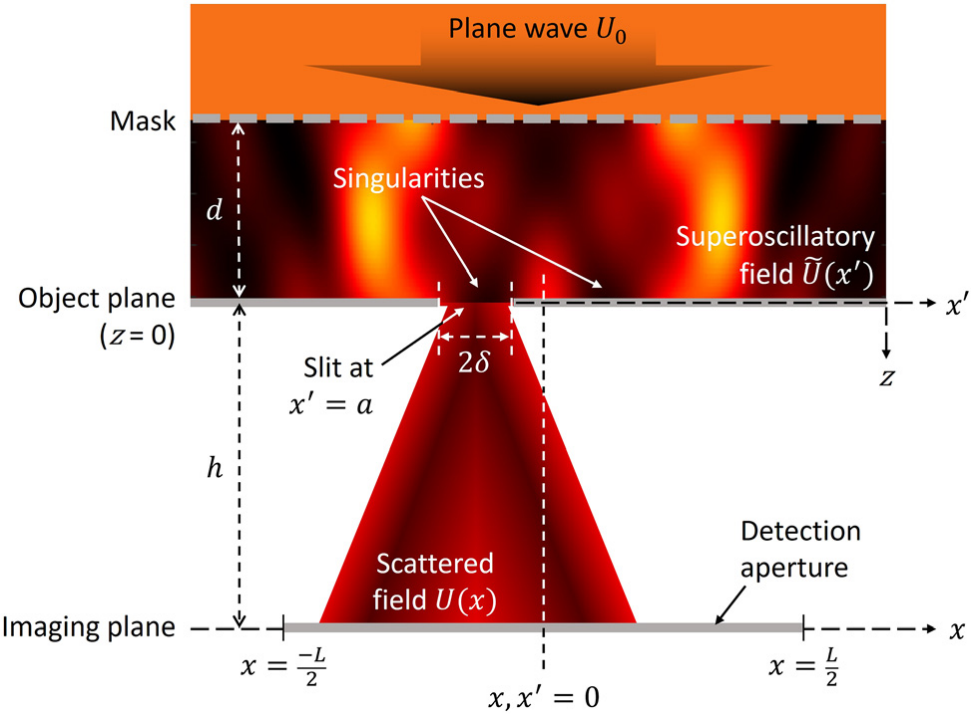
Scattering of a topologically structured field by a slit in an opaque screen. A plane wave *U*_0_ is incident upon a phase and amplitude mask, which generates a superoscillatory field $\tilde{U}\left(x^{\prime }\right)$ in the object plane *x*′, with a central hotspot located at *x*′ = 0. A slit of width 2*δ* is centered at *x*′ = *a* in the object plane. The scattered intensity $I\left(x\right)=U\left(x\right)U{\left(x\right)}^{*}$ is measured in the imaging plane *x*, at a distance *h* = 4*λ* beyond the object plane, with the imaging plane section −L/2<x<L/2 being projected onto a detector.

As a target object, we consider a slit of width 2*δ* = *λ*/10 in an opaque film (Figure 2). We assume that measurements are performed by analyzing its scattering pattern in an imaging plane located at a distance *h* = 4*λ* from the slit. From a practical perspective, the image sensor (detector) does not have to be at the imaging plane: the scattered field at this point is formed of free-space propagating waves, so it can be transformed to the detector plane by a conventional lens at any magnification, without loss of resolution (as has been shown experimentally [35], [42]). In what follows, we assume an imaging plane detection aperture at $-\frac{L}{2}<x<\frac{L}{2}$ , where *L* = 12*λ* (≫*δ*, *a*).

## 4 Fisher information analysis

To quantify and compare the effectiveness of localization metrology with different incident fields, we adopt the Fisher information metric, which quantifies the amount of the information that an observable variable carries about an unknown parameter (upon which the observable depends) [43]: A measurement that changes significantly in response to small changes in the unknown parameter provides a high amount of information about that parameter; Fisher information links to achievable measurement precision through the Cramér–Rao lower bound – the reciprocal of Fisher information being a lower bound on the variance of the unknown parameter. For example, in microscopic methods based upon localization of fluorescent molecules, Fisher information is related to the point-spread-functions obtained during measurements and can be used as a tool for their optimization [44], [45]. In quantum metrology, Fisher information can be used to fundamental limits applicable to parameter retrieval problems such as resolving incoherent point sources [46], time-varying waveform estimation [47], and quantum imaging [48], among others [49]. More recently, the Fisher information approach has been used to analyze optical scattering problems [50], [51] and as an optimization tool for scattering-based parameter estimation [52], [53], [54].

In the present case, we calculate Fisher information from the normalized intensity distribution at the image plane, for a given slit position, *a*:

$$p\left(x;a\right)=\frac{U\left(x,a\right)U{\left(x,a\right)}^{*}}{\overset{+L/2}{\underset{-L/2}{\int }}U\left(x,a\right)U{\left(x,a\right)}^{*}dx}$$

From a measurement perspective, this distribution and the relative rate of change in its log-likelihood function are important: The latter is known as the score function; the weighted square of which, integrated over the detection range *L*, is the Fisher information per measurement (photodetection event) - a figure of merit, in the present case, for how rapidly the profile of the scattered field changes in response to a change in the position *a* of the slit:

$$F\left(a\right)=\overset{+\frac{L}{2}}{\underset{-\frac{L}{2}}{\int }}{\left[\frac{\partial }{\partial a}\ln p\left(x;a\right)\right]}^{2}p\left(x;a\right)dx$$

**Figure 3:**
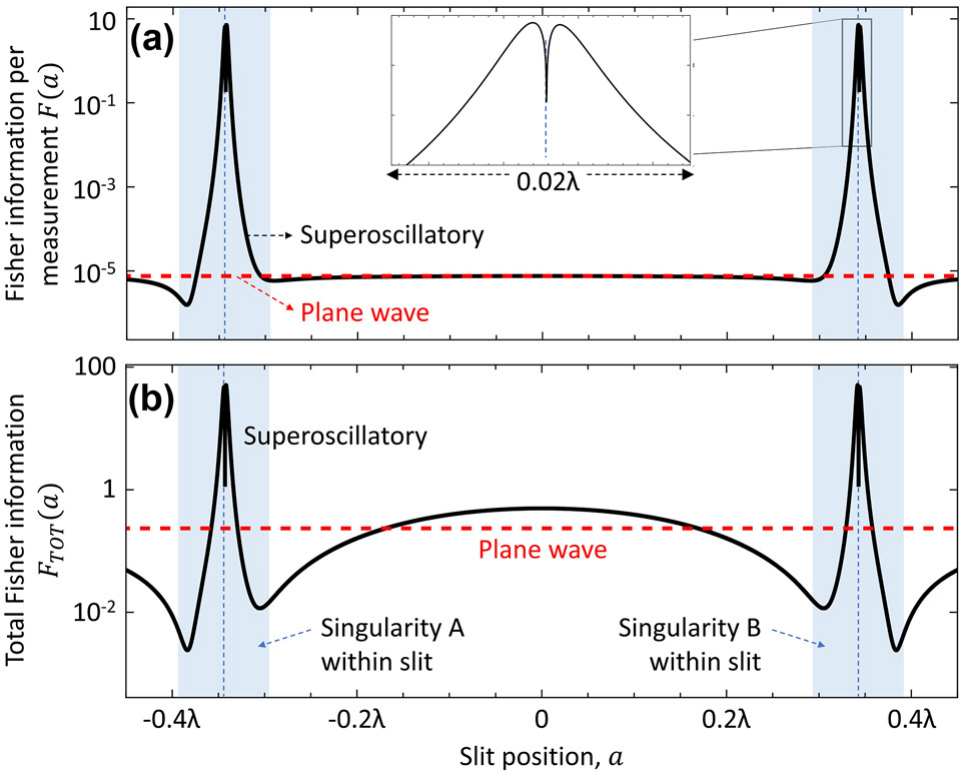
Fisher information content of the field scattered by a subwavelength slit. (a) Fisher information per measurement and (b) total Fisher information as functions of slit position for a superoscillatory incident field with amplitude $\left[21.65S_{2}\left(x^{\prime }\right)+S_{2}\left(x^{\prime }\right)\right]W$ – shown as solid black lines; a plane wave incident field with an intensity equal to half that of the superoscillatory hotspot – shown as dashed red lines. The blue shaded bands denote the range of positions over which a singularity (A or B, as labeled in Figure 1) is located within the slit.

Figure 3a shows that the Fisher information content of a scattered superoscillatory field depends strongly on the position of the scattering object within the incident field – in this case, most prominently on the position of the slit relative to the phase singularities. Note that, as long as the detector captures the entire scattered field, there is no dependence of Fisher information on slit position for an incident plane wave, because while the position of the diffraction pattern in the imaging plane shifts with the slit position in the object plane, its intensity profile is invariant. For the superoscillatory field, the Fisher information increases sharply, peaking at *a* = ±0.34*λ*, when either singularity A or B (on either side of the incident field’s central hotspot – see Figure 1) is near the center of the slit. The double- or split-peak structure, shown in the Figure 3a inset, is the result of a saddle point in the profile of the scattered field when the slit is centered on the singularity, whereby the scattered field is less sensitive to changes in slit position than for off-center alignments. At the peaks, the intensity profile of the diffraction pattern changes rapidly as a function of *a*, yielding a 10^6^-fold enhancement in Fisher information (Figure 3a), as compared to the plane wave from which the superoscillatory field was generated.

The fact that incident intensity is lower in the vicinity of phase singularities than in a plane wave, must be considered in this comparison: Figure 3b demonstrates that using a topologically structured incident field – i.e., probing the target object with an incident field containing singularities – nonetheless provides significant advantage, with the total information content in the detected scattered field:

$$F_{\text{TOT}}\left(a\right)=F\left(a\right)\int_{-\frac{L}{2}}^{+\frac{L}{2}}U\left(x,a\right)U{\left(x,a\right)}^{*}dx$$

still being enhanced by a factor of up to ∼250 when a singularity interacts with the slit, again as compared to the plane wave from which the superoscillatory field was generated.

It is also important to account for the fact that measurements at low intensities near a singularity are more susceptible to noise. Within the framework of Fisher information, this can be considered as follows: Each scattered field measurement (photodetection event) provides information $F\left(a\right)$ , while (detector) noise-related photodetection events provide zero information. Fisher information is additive, so in the presence of noise it is proportional simply to the ratio of scattered field detection events to all detection events. In terms of scattered field and noise intensities (*I* and *I*_*noise*_):

$$F\left(a,I_{\text{noise}}\right)=\overset{+\frac{L}{2}}{\underset{-\frac{L}{2}}{\int }}{\left[\frac{\text{d}}{\text{d}a}\ln\left(p\left(x;a\right)\right)\right]}^{2}p\left(x;a\right)\frac{I\left(x;a\right)}{I\left(x;a\right)+I_{\text{noise}}}dx$$

**Figure 4:**
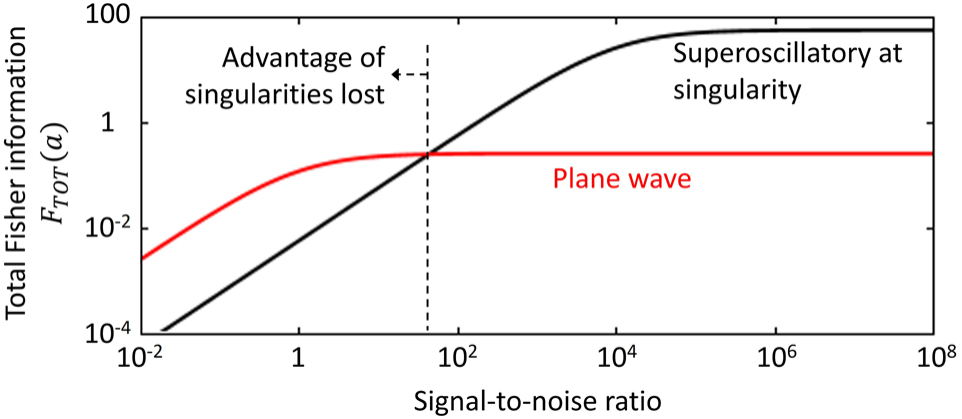
Total Fisher information as a function of signal-to-noise ratio for a superoscillatory incident field (assuming the slit to be optimally located at a singularity) [black line] and for a plane wave [red line].

Figure 4 shows total Fisher information as a function of signal-to-noise ratio (SNR). For consistent comparison, we assume the same plane wave intensity as used for generation of the superoscillatory field, and the same level of absolute noise in both cases. At high SNR (>1,000), the advantage of the superoscillatory incident field is obvious: Fisher information is orders of magnitude higher than for the plane wave. With decreasing SNR, the information content of the scattered field falls faster for the superoscillatory field, and the advantage is lost at signal to noise ratios <50.

## 5 Conclusions

In summary, this study provides a fundamental explanation, and justification, for singularity-based metrology. We demonstrate that when probing a nanoscale object, a significant advantage can be gained from exploiting phase singularities in a topologically structured incident field, as compared to plane wave illumination. Despite the low intensity in the vicinity of singularities, and in the presence of detector noise, Fisher information can be orders of magnitude larger when an object is illuminated with a topologically structured field containing phase singularities, as opposed to a plane wave. We show that this advantage – seen experimentally in the form of enhanced measurement precision and accuracy [35], [36] – is derived from the dependence of scattered intensity profile on local intensity and phase variations in the incident field at the object plane: small, deeply subwavelength changes in the position of a scattering object relative to a singularity can lead to large changes in the scattered field. The results presented here indicate that the optimization of the intensity and phase profiles of incident light according to the shape class and size range of objects can afford considerable enhancements of resolution in measurements with scattered light.
